# Cost-Effective Fabrication of Fractal Silicon Nanowire Arrays

**DOI:** 10.3390/nano11081972

**Published:** 2021-07-31

**Authors:** Antonio Alessio Leonardi, Maria José Lo Faro, Maria Miritello, Paolo Musumeci, Francesco Priolo, Barbara Fazio, Alessia Irrera

**Affiliations:** 1 Dipartimento di Fisica e Astronomia “Ettore Majorana”, Università di Catania, Via Santa Sofia 64, 95123 Catania, Italy; antonio.leonardi@dfa.unict.it (A.A.L.); mariajose.lofaro@dfa.unict.it (M.J.L.F.); Paolo.musumeci@ct.infn.it (P.M.); francesco.priolo@ct.infn.it (F.P.); 2 CNR-IPCF, Istituto per i Processi Chimico-Fisici, Viale F. Stagno D’Alcontres 37, 98158 Messina, Italy; 3 CNR-IMM UoS Catania, Istituto per la Microelettronica e Microsistemi, Via Santa Sofia 64, 95025 Catania, Italy; maria.miritello@infn.ct.it

**Keywords:** silicon nanowires, MACE metal-assisted chemical etching, fractal, photonics, erbium

## Abstract

Silicon nanowires (Si NWs) emerged in several application fields as a strategic element to surpass the bulk limits with a flat compatible architecture. The approaches used for the Si NW realization have a crucial impact on their final performances and their final cost. This makes the research on a novel and flexible approach for Si NW fabrication a crucial point for Si NW-based devices. In this work, the novelty is the study of the flexibility of thin film metal-assisted chemical etching (MACE) for the fabrication of Si NWs with the possibility of realizing different doped Si NWs, and even a longitudinal heterojunction p-n inside the same single wire. This point has never been reported by using thin metal film MACE. In particular, we will show how this approach permits one to obtain a high density of vertically aligned Si NWs with the same doping of the substrate and without any particular constraint on doping type and level. Fractal arrays of Si NWs can be fabricated without any type of mask thanks to the self-assembly of gold at percolative conditions. This Si NW fractal array can be used as a substrate to realize controllable artificial fractals, integrating other interesting elements with a cost-effective microelectronics compatible approach.

## 1. Introduction

Silicon nanowires (Si NWs) are emerging as a promising resource in different fields such as electronics [1,2,3,4,5], photovoltaics [6,7,8,9,10], and recently, photonics [11,12,13,14,15,16] and sensing [17,18,19,20,21]. Indeed, 1D nanostructures couple the nanomaterials’ advantages with a fabrication compatible with the typical flat architectures of the silicon industry. The approaches used for Si NW realization have a crucial impact on the final performances in all these application fields and determine the final device cost [22]. This makes the research on a novel and flexible approach that can push the Si NW performance a crucial point for Si NW-based devices. Some of the most used approaches are Vapor–Liquid–Solid (VLS) and Deep Reactive Ion Etching (DRIE). However, both of them present several issues that can detrimentally affect the performance and cost of Si NWs. 

By using VLS, it is very complex to obtain highly oriented NWs with diameters under tens of nanometers, and, due to this this limitation, disordered bunches of nanowires are commonly synthesized [23,24]. The need for high process temperatures is another strong drawback that causes the occurrence of metal contamination and doping disuniformity (when doping is carried out during the growth). DRIE is an anisotropic dry etching that requires a masking procedure. Whereas Electron Beam Lithography (EBL) is not suitable for large-scale production, in recent years, other lithography strategies have emerged. An example is nanoimprinting lithography (NIL) that shows similar EBL features but couples large-scalable fabrication. However, the high cost, the need for expensive dedicated equipment, and the limit of 50:1 (in some state-of-the-art cases, up to 100:1) on the aspect ratio affect this approach for the realization of ultrathin NWs.

All these issues characterize the demand for novel flexible fabrication methods that can allow a cost-effective realization of high-quality Si NWs with great control on doping and in-plane geometry. In recent years, metal-assisted chemical etching (MACE) arose as an innovative anisotropic wet etching method able to promote a cost-effective, large-scalable, and microelectronics-compatible fabrication [22,25,26]. In this approach, highly electronegative metals are used to catalyze and drive the Si etching at room temperature. While masked approaches on the metal structure permit one to achieve a high control on the Si structure geometry, the mask resolution limits the diameter that can be obtained, thus making the fabrication of quantum confined Si NWs very complicated. Moreover, all these approaches add another commonly complex and expensive step to the synthesis. Different authors [27,28,29,30] demonstrate the application of maskless metal film MACE. An alternative route to ordered material that found interesting applications in several fields is represented by disordered nanostructures, by a far less expensive and easy-to-fabricate design.

Moreover, different textural disorder approaches are emerging in novel materials made up of arrays of nanostructures, such as fractal disorder. This is due to a variety of remarkable phenomena that can arise in these media such as superconductivity, superdiffusivity, and non-linear optics. Moreover, fractals have interesting applications in photonics. Indeed, their ability to promote strong multiple scattering and trap light in a wide wavelength range is potentially useful in developing next-generation optical devices [14]. In the photovoltaics field, this occurrence becomes of crucial interest to reduce the cost of the produced energy [6]. All these aspects have driven the realization of a 2D random fractal of Si NWs with the extreme advantage of being compatible with the current Si industry [14,31].

Our group has demonstrated the realization of a 2D random fractal array of Si NWs by using a fractal thin metal film of a few nanometers of Au as a catalyst. In two-step MACE, the Si NW array is obtained as the negative mask of the starting discontinuous metal film. In particular, the metal acts as a catalyst driving the silicon etching underneath the film, while the uncovered silicon is unetched and will form the Si NWs. Hence, the engineering of the Au film can be an interesting strategy to texture the Si NW array. In the literature, it is well known that a percolative gold layer is a 2D random fractal [32]. A percolative gold layer is characterized by the appearance (or disappearance) of connected regions of an infinite extent that permit the constant identification of a path connecting the edges of the considered area [33]. The percolation threshold is directly correlated to a fractal geometry of the system and it is well known that by using a percolative gold layer deposition, it is possible to obtain a fractal Au film. By the self-assembly of a thin gold layer in percolative conditions, this method permits one to obtain both room temperature light-emitting Si NWs and fractal in-plane geometry [14,31]. Contrary to most of the literature reporting NWs with a diameter of several tens of nanometers, our approach permits the fabrication of sub-10 nm thick NWs and a huge density of 10^12^ NWs/cm^2^. The use of the self-assembly of gold is a cost-effective solution for a controllable morphology that does not imply the use of any masking approach. Indeed, in our case, no masking procedures are applied, and the NW geometry is determined by the negative development of the percolative gold layer. In our work, we demonstrate that the interesting characteristics of a cost-effective, fast, industrially compatible, and flexible synthesis of MACE can be coupled with a quantum confinement suitable dimension and fractal array organization by our synthesis.

The interest in 2D artificial random fractal Si NWs has driven our research through new methods to fabricate controllable artificial fractals based on our Si NWs integrated with other interesting materials. Among all the elements that can be coupled with Si, the integration of different light-emitting materials represents an interesting opportunity for silicon photonics [13,34,35]. In particular, erbium has a special role as one of the most studied and interesting elements that is compatible with standard silicon technology [36,37,38]. However, Er is characterized by low solubility and low excitation cross-section in a silicon substrate [39,40,41,42], and an interesting strategy to overcome these issues involves the use of erbium silicates. In these materials, erbium is not a dopant but a constituent [43]. Among them, yttrium oxide (Y_2_O_3_) is a Si-compatible material [44] that arises as a strategic solution to minimize the ion–ion interactions [45] that may suppress the Er light emission. Indeed, Y_2_O_3_ and Er_2_O_3_ crystalline structures are very similar and Er atoms can be introduced in the Y substitutional position. In our approach, we demonstrated the possibility to integrate Er in an industrially compatible way in Si NW arrays without any damaging process and without the need for further post-processing approaches. Moreover, this proposed glancing angle decoration process, coupled with the fractal Si NW structure, permits one to control the optical properties by simply varying the deposition angle [14]. In contrast to ionic implantation, where it is necessary to change the implant dose, in our case, this does not introduce any further defects and further processes, such as annealing, are not required.

In this paper, we report for the first time the thin film MACE synthesis of Si NWs with different doping and with the possibility of realizing longitudinal p-n heterojunction inside the same wire. This point, coupled with the extreme flexibility of our approach to the realization of Si NWs, has never been reported. A comprehensive overview of the main properties of Si NWs realized by thin-film MACE is presented. In particular, an important novelty is the study and characterization of the NW length and etching velocity of the thin film MACE as a function of the doping level, thus highlighting how this approach allows one to obtain NWs of different doping types and levels, even permitting the growth of p-n-doped Si NWs during the same etching procedure. Concerning the texture and in-plane geometry, it is shown how, without any expensive mask, this approach can be carried out for the realization of 2D random fractal arrays. These 2D fractal Si NWs can be used as substrates in a glancing angle sputtering deposition for the realization of controllable artificial fractal arrays in silicon and with a cost-effective microelectronics-compatible approach that can integrate other interesting elements. As an example of that, we report the decoration of fractal Si NWs with Er:Y_2_O_3_. The angle at which the deposition is performed can be used to vary and control the final fractal morphology, showing an interesting perspective for disorder engineering in a silicon platform.

## 2. Materials and Methods

### 2.1. Materials and Chemicals

Hydrofluoric acid was bought from Honeywell, while hydrogen peroxide was purchased from Sigma Aldrich (Merck KGaA Headquarters of the Merck Group, Frankfurter Strasse 250, Darmstadt, Germany). High-purity (99.9%) gold pellets as the Au source for electron beam evaporation and the aluminum zinc oxide (AZO) target for sputtering deposition were acquired from Kurt J. Lesker (Kurt J. Lesker Company 1925 Route 51 Jefferson Hills, PA 15025 USA). Si NWs were synthesized starting from a 4” (100)-oriented Si wafer acquired from Siegert Wafer (SIEGERT WAFER GmbH, Charlottenburger Allee 7, Aachen, Germany).

### 2.2. Thin Film Metal-Assisted Chemical Etching and AZO Deposition

Si NWs were fabricated by the thin film metal-assisted chemical etching (MACE). This method involves anisotropic wet etching catalyzed by a discontinuous few-nanometer metal film. Firstly, the Si wafers with different doping types were cleaned and the native silicon oxide was removed by HF etching with a 2.5 M hydrofluoric acid aqueous solution. The samples were then inserted inside the high vacuum chamber of an electron beam evaporator and a 2 nm thick discontinuous gold film was deposited at room temperature and in a high vacuum condition (<10^−6^ mbar). After the deposition, the samples were immersed in an etching solution of 5 M HF and 0.44M H_2_O_2_. The gold acted as a catalyst, inducing the local oxidation (favored by the H_2_O_2_) of the silicon in contact with the metal. The SiO_2_ formed underneath the gold was then dissolved due to the presence of the HF. As a consequence, the Si was anisotropically etched just underneath the gold and formed the NWs in the uncovered regions. Finally, Au was removed by a gold etching solution and, since all the processes were at room temperature, no gold contamination occured. 

### 2.3. Characterization Equipment and Measurement Details

Scanning Electron Microscope (SEM) images were acquired by a ZEISS SUPRA 25 (Carl-Zeiss-Straße 22, Oberkochen, Germany). The fracLac plugin of ImageJ analysis software through the non-overlapping box-counting algorithm was used to measure the fractal parameters of fractal dimension and lacunarity. The algorithm was implemented on plan-view SEM images acquired at a magnification of 50 kX and 100 kX. During the approach, the images were sectioned in a grid of variable dimensions measuring the pixel density statistic (average value and standard deviation) for each box dimension to calculate both fractal dimension and lacunarity.

The Er:Y_2_O_3_ film thickness was measured by both SEM analysis and Rutherford backscattering spectrometry (RBS) on the Si bulk reference substrates. RBS analyses were also carried out to measure the elemental composition of Er:Y_2_O_3_-decorated Si and Si NWs. In all the RBS experiments, a He^+^ beam at an energy of 2 MeV impinged onto the samples and the backscattered He^+^ ions were collected at the detection angle of 165° with respect to the beam direction. The energy loss of the backscattered ions was measured by a multichannel analyzer. Er silicates’ presence was investigated by X-ray diffraction (XRD) measurements performed onto Si bulk and Si NWs by using a Bruker X-Ray Diffractometer.

## 3. Results and Discussion

Si NWs were synthesized according to the thin film MACE process as described in the Materials and Methods section. A 2 nm discontinuous Au layer was deposited on a Si substrate by electron beam evaporation. Subsequently, the sample was immersed in an aqueous solution of hydrofluoric acid and hydrogen peroxide. The Si etching was driven by the gold film and the Si was unetched in the uncovered Si regions where Si NWs were realized.

**Figure 1:**
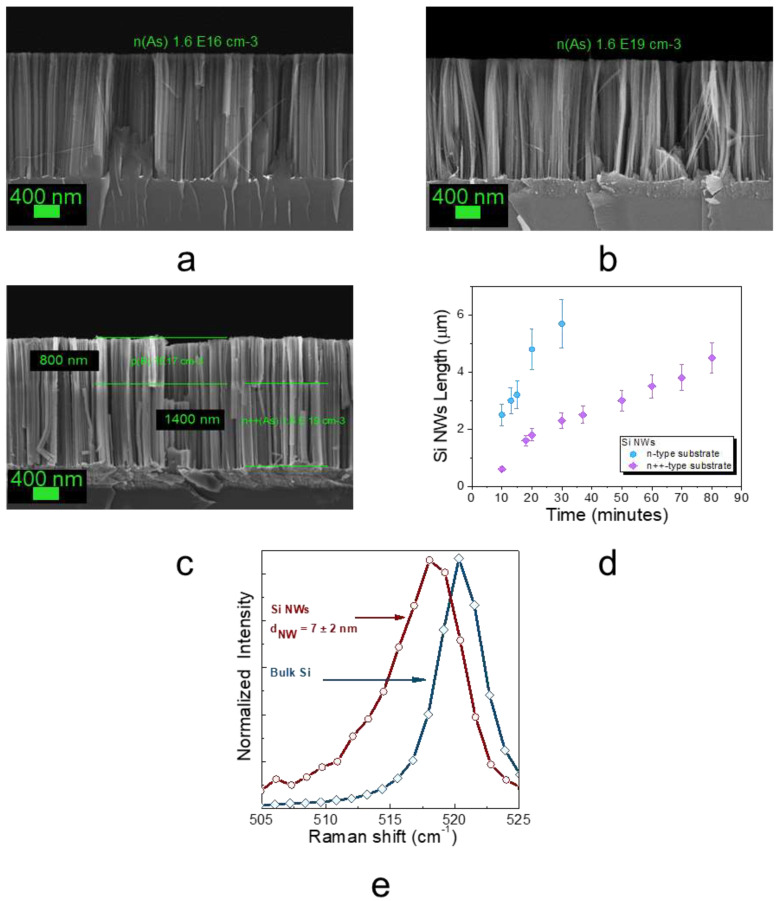
Cross-section SEM images of Si NWs realized starting from lightly n-doped (As atoms 1.6 × 10^16^ cm^−3^, resistivity 5 Ohm × cm), heavily n-doped (As atoms 1.6 × 10^19^ cm^−3^, resistivity 0.005 Ohm × cm), and p-n (B atoms 1 × 10^17^ cm^−3^, resistivity 0.5 Ohm × cm and As atoms 1.6 × 10^19^ cm^−3^, resistivity 0.005 Ohm × cm) substrates in (**a**–**c**) respectively. (**d**) Si NW length as a function of the etching time for lightly doped (indicated as n-type) and heavily doped n-type (indicated as n++-type) substrates (**e**).

In Figure 1a,b, the cross-section Scanning Electron Microscope (SEM) images of two Si NW samples obtained starting from two n-doped substrates with As concentrations of about 1.6 × 10^16^ cm^−3^ (resistivity 1–5 Ohm × cm) and 1.6 × 10^19^ cm^−3^ (resistivity 0.005 Ohm × cm), are, respectively, reported. Indeed, by this synthesis method, the Si NW doping is the same as the starting Si substrate, and by changing the substrate doping, it is possible to obtain different doped NWs. This approach is extremely flexible and, when using a p-n-doped Si substrate, it is even possible to obtain simultaneously p-n-doped Si NWs. Starting from a Si substrate with an about 800 nm p-doped (B atoms 1 × 10^17^ cm^−3^, resistivity 0.5 Ohm × cm) layer on the remaining heavily n-doped (As atoms 1.6 × 10^19^ cm^−3^, resistivity 0.005 Ohm × cm) bulk, we obtained Si NWs with a p-doped region in the first 800 nm of layer thickness and an n-doped one in the remaining 1400 nm (as reported in Figure 1c). As sketched in Figure 1c, this approach permits us to realize large-scale high-density Si NWs with a p-n junction inside each NW. All the reported Si NWs are about 2.2 µm long and with a huge Si NW density of about 10^12^ NWs/cm^2^ [22], a crucial point for all the applications.

By changing the etching time, it is possible to vary the Si NW length by using (100)-orientated wafers in the case of n-type doping, as shown in Figure 1d. In particular, the length of the obtained Si NW lightly n-doped (As atoms 1.6 × 10^16^ cm^−3^, resistivity 1–5 Ohm × cm) and heavily n-doped (As atoms 1.6×10^19^ cm^−3^, resistivity 0.005 Ohm × cm) are reported as a function of the etching time in cyan and pink, respectively. In standard MACE processes, the etching velocity strongly depends on the doping type and level [46], and in our fabrication approach, we found this etching velocity to be very similar for lightly doped p and n NWs [11]. Indeed, the etching process occurs following the Si oxidation that is driven by the hole injection catalyzed by the metal. In particular, the hole injection depends on the redox potentials of the etch solution (catalyzed by the metal) and on the Fermi level E_f_ of the semiconductor [47,48,49] that is determined by the doping. In particular, here, we show that as the doping level increases from the slightly doped substrate to the heavy one, there is a decrease factor of 2.5 in the etching ratio (expressed as length/time).

Raman spectroscopy is a very diffused and investigated approach for the study of Si-based nanostructures [50], able to provide interesting information. In Figure 1e, the first-order Stokes Raman signal of the Si NWs and of Si bulk is shown in red and blue, respectively. The Si NWs signal is characterized by an asymmetrically broadened peak red, shifted with respect to the symmetric and sharper signal typical of bulk crystalline Si, which is found at 520 cm^−1^. The shift and the asymmetrical shape are in agreement with the literature concerning quantum confined crystalline Si nanostructures. Indeed, this peak can be fitted by the Campbell and Fauchet [51,52,53] phenomenological method to obtain a NW diameter value of 7 ± 2 nm for the 2 nm thick Au film.

The main structural parameters of these fabricated Si NWs are reported in Table 1 compared to the literature.

**Figure 2:**
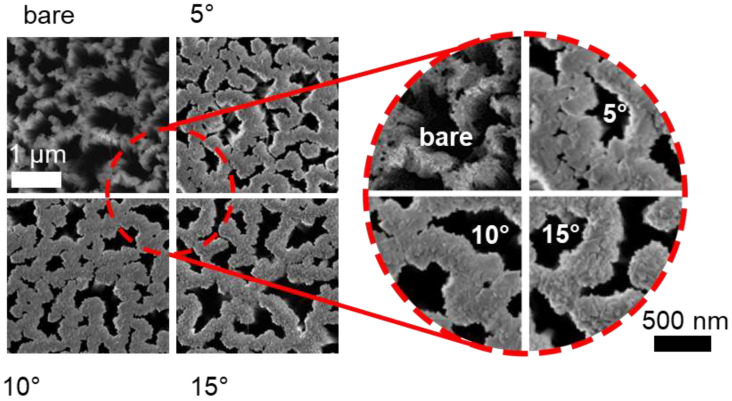
Plan-view SEM images of fractal bare Si NWs, 5°, 10°, and 15° Er:Y_2_O_3_ decorated at the same magnification are reported on the left side. On the right side, to highlight the different coverage and filling factor, a magnification of all these images is shown.

Another crucial point, discussed in the Introduction, is that this fabrication method allows the realization of fractal arrays of Si NWs that can be decorated with other materials for the realization of Si-based artificial fractal. In Figure 2, the as-grown fractal Si NW array, as well as the decorated samples with Er:Y_2_O_3_ at 5°, 10°, and 15°, are shown. It is clearly visible that the decoration process does not close all the air gaps. This is a crucial point that preserves and engineers the fractal morphology of the system. Indeed, the interstices among Si NWs appear to be more covered for small angles, with a clear variation of the fill factor (FF). The fill factor is the ratio of the covered area per the total area of the analyzed image that we measured by pixel counting with the ImageJ software. An FF of 42 ± 2% was measured for the bare Si NWs sample, while an FF of 76 ± 1%, 74 ± 1%, and 69 ± 1% for Er:Y_2_O_3_-decorated Si NWs at 5°, 10°, and 15° angles were obtained. The higher the angle, the smaller the measured FF, with a decrease in the in-plan coverage. This effect can be understood considering that higher deposition angles correspond to the smaller aperture angle of the shadowing cone. Therefore, by varying the oblique angle used in the sputtering process, we can tune the fractal morphology of the decorated samples. Indeed, the fractal key feature is the scale invariance that can be measured as the scale invariance of the FF at different magnifications [14,61]. Both the bare Si NWs and the Er:Y_2_O_3_-decorated Si NWs show a constant fill factor at different magnifications (10–300 kX range). 

The fractal nature of the sample was studied by the plugin FracLac sliding box of ImageJ used on the binary converted SEM images. The sliding box algorithm parameters were set as equal for all the samples to guarantee consistency between the results. Both fractal dimension and lacunarity were investigated for each deposition angle. Lacunarity is a parameter introduced by Mandelbrot [62] that takes into account the statistical fluctuation of the mass probed by the box-counting algorithm for each fixed dimension of the boxes. Lacunarity provides complementary information to the fractal dimension, describing how the fractal texture is organized in terms of the heterogeneity of the sample represented by the fluctuation of the distribution of the alternation of empty and filled spaces as a function of the length scale [63]. As it is possible to observe by the magnification of the SEM images in the right part of Figure 2, the deposition angle affected the FF and the heterogeneity of the samples. These cause different lacunarities at different deposition angles. Therefore, with a simple and cost-effective approach, it is possible to realize artificial fractals based on Si NWs where the morphological and structural fractal parameters can be tuned by varying the deposition angle. By controlling the mass fluctuation in terms of lacunarity, it is possible to tune the refractive index fluctuation, and this result is interesting for photonics and photovoltaics, opening the possibility of tuning the light-scattering properties of the system.

**Figure 3:**
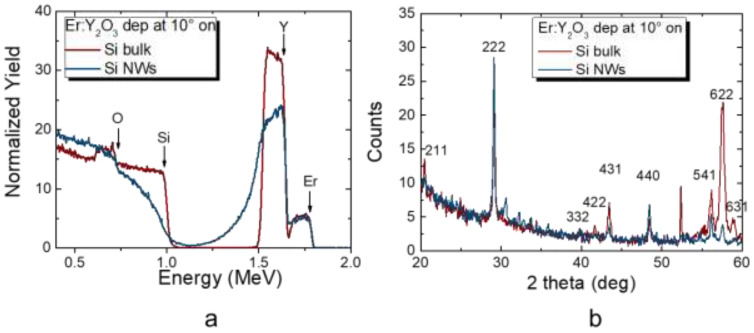
(**a**) Rutherford backscattering spectrometry of the decorated Si bulk (red) and Si NWs (blue) obtained at 10°. (**b**) X-ray diffraction analysis of the Er:Y_2_O_3_ deposited on Si bulk and Si NWs at 10°.

The stoichiometry of the elemental composition of the Er:Y_2_O_3_-decorated sample was confirmed by means of a Rutherford backscattering spectrometry (RBS) analysis, shown in Figure 3. Fractal Si NWs are characterized by the presence of air gaps of sizes spanning in the nm-μm range [64,65]. In Er:Y_2_O_3_-covered Si NWs, the air gaps between the wires are reduced but still present at all the length scales (otherwise, there would be no presence of a fractal). This strong discontinuous structure makes the fitting and analytical interpretation of RBS data extremely complex (NW signal in blue in Figure 3a). As it is observed in Si NW samples, the signals of the constitutional elements (such as Si and Y) are strongly influenced by the discontinuity of the substrate. Indeed, a He^+^ ion can be backscattered from the NW tips as well as from the Si substrate (at 2.3 μm of difference, considering the NW height). This strong variation in the Si atom position is reflected in the RBS spectrum shape. These critical issues motivate the stoichiometry quantitative analysis on Si bulk (in red in Figure 3a). Indeed, it is expected for the 5°, 10°, and 15° Er:Y_2_O_3_ depositions on Si bulk to obtain the same stoichiometry at the same deposition conditions of the Si NW samples. In Figure 3a, the signal obtained from the Er:Y_2_O_3_ on Si bulk sample is shown in red (10° deposition). The presence of the Er, Y, Si, and O signals is highlighted in the graph. The position of these peaks and their extension were fitted by the simulation software SIMNRA, which found an Er concentration of 2 ± 1 at% (areal density of about 0.3 × 10^17^ at/cm^2^), while Y and O showed a concentration of about 38 ± 2 at% (areal density of 4.5 × 10^17^ at/cm^2^) and 60 ± 5 at% (areal density of 7 × 10^17^ at/cm^2^), respectively. For the Er:Y_2_O_3_-decorated samples (blue spectrum), the Si signal shows a clear shift towards lower energy loss channels, which is the result of the additional energy loss of the ion probe due to the Er:Y_2_O_3_ concentration onto the surface.

It is worth noticing that after the Er:Y_2_O_3_ decoration, both the decorated Si bulk and Si NWs attest about the same downshift, suggesting the same concentration of Er, Y, and O deposited on the two substrates, as expected. This point is strongly confirmed by the same Er and Y integrated peak in the two samples that indicate the presence of the same total amount of material. After the RBS analysis, the Si NWs (in blue) and Si bulk (in red) decorated samples were characterized by XRD with a Bruker X-Ray Diffractometer to demonstrate the absence of Er silicates between Er:Y_2_O3 and Si NWs. It is well known that the presence of Er silicates at the interfaces can strongly detrimentally affect the PL emission, acting as non-radiative de-excitation channels. In Figure 3b, the XRD spectra of the Si bulk and Si NWs decorated at 10° (reported as an example) confirm the good crystalline quality of the Er:Y_2_O_3_ films. The fingerprint of the cubic Y_2_O_3_ crystalline structures is visible for the two samples, and the characteristic X-ray diffraction peaks of silicates are not visible, demonstrating their absence. This result is in agreement with the literature, where the formation of Er silicate at the interface between Er:Y_2_O_3_ and Si NWs is absent at a deposition substrate temperature of 300 °C [66]. 

## 4. Conclusions

In this paper, the novelty reported is the flexibility of the thin film MACE for the realization of Si NWs and the study of Si NWs characterized by different doping types, and a longitudinal p-n heterojunction inside the same wire. In particular, this approach permits the fabrication NWs in tens of minute, with length up to few microns, without constraints on the doping of the wires that is determined by the starting substrate. Moreover, for the first time, we have demonstrated that by using a wafer with two different doping types, it is even possible to realize p-n NWs with a large-scalable, cost-effective, and microelectronics-compatible approach. To further show the possibility offered by this approach, the fabrication of novel artificial 2D fractal arrays based on Er:Y_2_O_3_-decorated Si NWs was reported. In particular, the use of glancing angle sputtering was successfully adopted to decorate fractal Si NWs, enabling a fractal morphology control. The Er concentration and the consistency between the different sputtering processes were confirmed through Rutherford backscattering spectrometry, and the absence of silicates was demonstrated by XRD. Thanks to the possibility to control the fractal and lacunarity properties in a glancing angle deposition, this result opens the route to the realization of novel and controllable artificial fractals that integrate interesting elements in a silicon microelectronics-compatible substrate.

## Figures and Tables

**Table 1:** Comparison between Si NWs in this work and literature.

Length (µm)	Average Diameter (nm)	Density (NW/cm^2^)	Fab. Time (min)	Doping	Other Char.	Method	Ref.
2–3	5–10	10^12^	10–50	n++-,n-type, p-n++ junction	Fractal arrays and quantum confined	2 nm Au MACE	This work
2–3	≥39	10^8^	/	Undoped	Si NWs inside microchannel structures	VLS	[23]
1.5–3	40–50	/	6–8	/	RIE coupled with nanoimprinting lithography	Deep RIE	[54]
5–20	70–100	/	20–60	p-type	Metal patterned area by UV lithography	Silver salt MACE	[55]
1.5–6	20–300	/	6–10	n-type	Metal patterned area by Colloidal templates	Au/Ti thin film MACE	[30]
1.5–2	60–120	10^8^–10^9^	5–10	n-type	Si NWs size further reduced by KOH etching with tailored shape design	Au film MACE	[56]
Up to 100	200–3000	/	60–180	p-, n-type	Microstructurres	Ag/Au film Mace	[57]
2–3	500–100	10^7^–10^8^	20	p-type	Tailored shape Si wires	Au/Ti thin film MACE	[58]
0.2–10	55	10^8^–10^9^	0.5–4	n-type	Metal patterned area by nanosphere lithography	Au thin film MACE	[59]
1	40–60	10^8^–10^10^	60	p-type	Metal patterned area by AAO membranes	Au/Ag thin film MACE	[60]

## Data Availability

Data are contained within the article.
